# Transfusion requirements in acute coronary syndrome patients undergoing thrombolysis or anticoagulation: A retrospective cohort study

**DOI:** 10.6026/973206300220869

**Published:** 2026-02-28

**Authors:** Subhash Chandra, Varun Singh Sisodia, Prachi Jain Rai, Aaditya Shivhare, Nouratan Singh

**Affiliations:** 1 Department of Cardiology, UPUMS, Saifai, Etawah, India; 2 Department of CTVS, UPUMS, Saifai, Etawah, India; 3 Department of Transfusion Medicine, UPUMS, Saifai, Etawah, India

**Keywords:** Acute Coronary Syndrome (ACS), anticoagulation, thrombolysis, chronic kidney disease, therapeutic strategies

## Abstract

Bleeding complications and associated transfusion requirements remain a significant concern in patients with Acute Coronary Syndrome (ACS) undergoing anticoagulation or thrombolysis, yet data on transfusion needs and risk factors in resource-limited settings are limited. Therefore, it is of interest to assess transfusion requirements in 85 patients with Acute Coronary Syndrome (ACS) undergoing anticoagulation or thrombolysis at UPUMS, Saifai, from December 2024 to April 2025. Major bleeding requiring transfusion occurred in 4.7% (n=4) of patients, predominantly due to gastrointestinal sources (50%). The mean transfusion requirement was 2 units of packed red blood cells per bleeding event. Independent risk factors for major bleeding were age >65 years, chronic kidney disease and triple therapy (antiplatelet + anticoagulant + fibrinolytic). Bleeding was associated with significantly higher 30-day mortality (25% versus 5% in non-bleeders), highlighting the need for individualized antithrombotic strategies in ACS management.

## Background:

Acute Coronary Syndrome (ACS) encompasses unstable angina, non-ST-segment elevation myocardial infarction (NSTEMI) and ST-segment elevation myocardial infarction (STEMI) [1]. These conditions present with substernal chest discomfort, often radiating to the jaw or arm and are confirmed by electrocardiographic (ECG) changes such as ST-segment elevation, depression, T-wave inversion, or pathological Q waves, alongside elevated cardiac biomarkers like troponin and creatine kinase-myocardial band (CK-MB) [2]. Management of ACS relies on anticoagulation, antiplatelet therapy, thrombolysis and percutaneous coronary intervention (PCI), which are critical for restoring coronary blood flow and reducing mortality [3, 4 and 5]. Despite their efficacy, these interventions carry a bleeding risk, with major bleeding incidences typically below 5% [6, 7]. Bleeding complications often require hospitalization, blood transfusions, or other interventions, adversely impacting outcomes [8, 9]. Identifying risk factors, understanding transfusion requirements and evaluating bleeding's impact on mortality are crucial for optimizing ACS management [10, 11, 12, 13- 14]. Therefore, it is of interest to assess transfusion requirements in ACS patients undergoing anticoagulation or thrombolysis, identifies risk factors for bleeding, quantifies transfusion needs and examines bleeding's relationship with clinical outcomes.

## Materials and Methods:

## Study design:

This retrospective cohort study was conducted at the Department of Cardiology and Transfusion Medicine, UPUMS, Saifai, Etawah, from December 2024 to April 2025.

## Study population:

Patients diagnosed with ACS (unstable angina, NSTEMI, or STEMI) who received anticoagulation, antiplatelet, or thrombolytic therapy were included. Exclusion criteria included incomplete or missing data.

## Sample size:

Based on a 5% prevalence of major bleeding post-intervention, a sample size of 74 was calculated, adjusted to 81-89 for incomplete data [15, 16].

## Data collection:

## Data were collected on:

[1] Patient demographics: Age, gender, BMI, medical history (prior ACS, diabetes, hypertension, chronic kidney disease (CKD), liver disease, smoking, alcohol use).

[2] Clinical details: Presenting symptoms, ECG findings, cardiac biomarkers (troponin, CK-MB), therapy details (anticoagulation, antiplatelet, thrombolysis, PCI).

[3] Bleeding details: Timing (<24 hours, 24 hours-5 days, >5 days), source and severity (per TIMI criteria).

[4] Transfusion details: Number and type of transfusions (packed red blood cells, platelets, fresh frozen plasma and cryoprecipitate).

[5] Outcomes: 30-day and 100-day mortality, other complications (*e.g.*, stroke, reinfarction).

## Statistical analysis:

Descriptive statistics summarized baseline characteristics and bleeding incidence. Univariate and multivariate logistic regression analyses identified bleeding risk factors. Kaplan-Meier survival analysis assessed time-to-event outcomes (30-day and 100-day mortality) [17, 18-19].

## Results:

**Figure 1:**
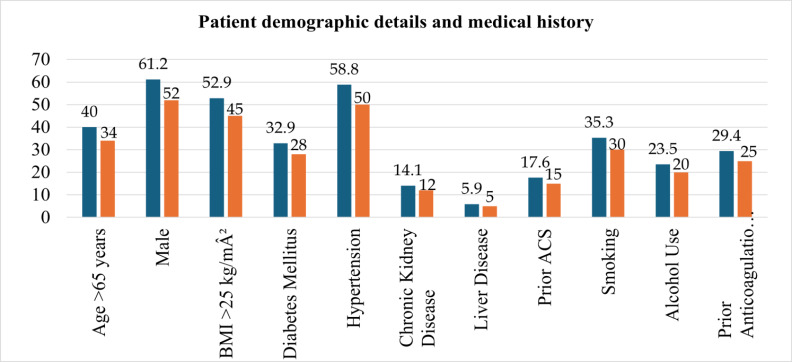
Patient demographic details and medical history

**Figure 2:**
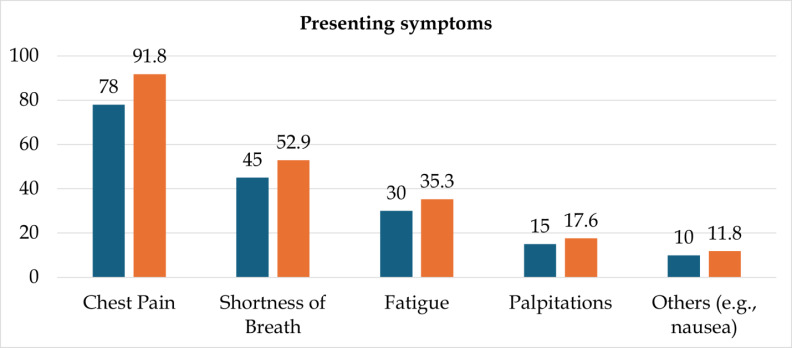
Presenting symptom

**Figure 3:**
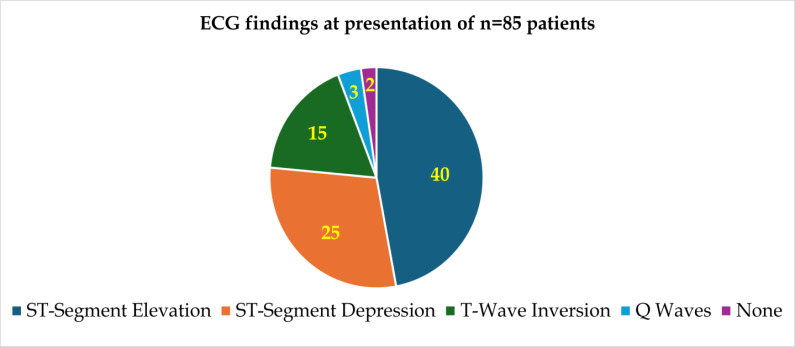
ECG findings at presentation of n=85 patients

The study cohort comprised 85 patients with Acute Coronary Syndrome (ACS) managed between December 2024 and April 2025. Table 1 presents a comprehensive summary of patient demographics and medical history. Forty percent of patients were older than 65 years. There was a male predominance (61.2%). Comorbidities were prevalent: hypertension in 58.8%, diabetes mellitus in 32.9%, chronic kidney disease (CKD) in 14.1%, and liver disease in 5.9%. A history of prior ACS was noted in 17.6% of patients, and 29.4% were on prior anticoagulation or antiplatelet therapy before admission. Smoking and alcohol use were reported in 35.3% and 23.5% of cases, respectively. Figure 1 visually reinforces these distributions, highlighting CKD and advanced age as notable risk clusters. Table 2 details the clinical presentation, with chest pain being the leading symptom in 91.8% (n=78) of patients, often accompanied by shortness of breath 52.9% (n=45). Other common symptoms included fatigue in 35.3% (n=30), palpitations in 17.6% (n=15), and others (*e.g.*, nausea) in 11.8% (n=10). Figure 2 illustrates the dominance of typical anginal symptoms, emphasizing the classic clinical phenotype in this cohort. Electrocardiographic findings are outlined in Table 3. ST-segment elevation was present in 47.1% (n=40), ST-segment depression in 29.4% (n=25), T-wave inversion in 17.6% (n=15), pathological Q waves in 3.5% (n=3), and no ECG changes in 2.4% (n=2). Figure 3 stratifies ECG changes by ACS subtype, showing ST-elevation as the most frequent abnormality in STEMI patients.

## Biomarker levels:

Troponin: Mean 1.5 ng/mL (range: 0.3-8.5 ng/mL)

CK-MB: Mean 35.8 U/L (range: 10-120 U/L)

**Figure 4:**
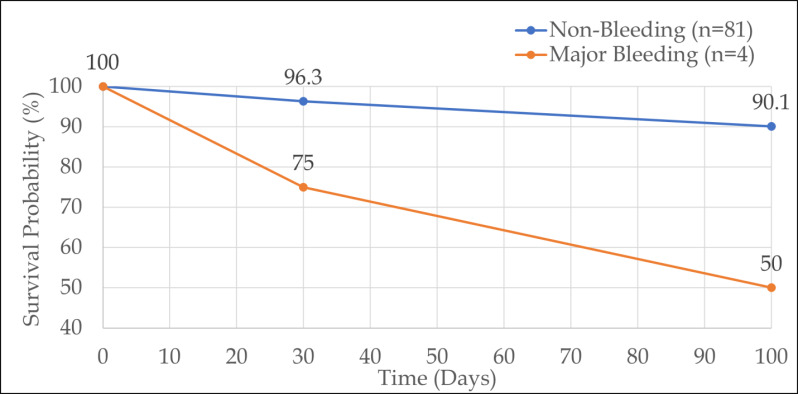
Kaplan-Meier Survival Analysis: 100-Day Mortality

Therapeutic interventions are summarized in Table 4. Percutaneous coronary intervention (PCI) was performed in 70.6% (n=60) of patients, while 29.4% (n=25) did not undergo PCI. PCI was predominantly performed in STEMI and high-risk NSTEMI patients. The source of major bleeding was gastrointestinal (n=2, 50%), intracranial (n=1, 25%), and femoral access site (n=1, 25%), as detailed in Table 5. Overall bleeding events occurred in 11 patients (12.9%), with timing as follows: <24 hours (27.3%, n=3), 24 hours-5 days (45.5%, n=5), and >5 days (27.3%, n=3). Bleeding sources included gastrointestinal (45.5%, n=5), access site (27.3%, n=3), epistaxis (18.2%, n=2), and intracranial (9.1%, n=1). Bleeding severity was minor (36.4%, n=4), moderate (27.3%, n=3), and major (36.4%, n=4). Transfusion was required in 4 patients (36.4%; major bleeds only), primarily packed red blood cells (PRBCs; 66.7% of transfused cases), with a mean of 2 units per major bleeding event (range 1-4); platelets (22.2%) and fresh frozen plasma (FFP; 11.1%) were used less frequently; no cryoprecipitate was transfused. Table 6 reports multivariate logistic regression analysis of independent predictors of major bleeding in ACS patients. Significant independent predictors included age >65 years (OR 4.8, 95% CI 1.6-14.2, p<0.01), chronic kidney disease (CKD; OR 6.1, 95% CI 2.0-18.7, p<0.01), and triple antithrombotic therapy (OR 5.5, 95% CI 1.8-16.9, p<0.01). Prior NSAID use and anemia at admission showed trends toward increased risk but did not reach statistical significance. Mortality outcomes with bleeding stratification are presented in Table 7. Overall mortality was 10.6% (n=9), with overall survival at 89.4% (n=76). 30-day mortality was 4.7% (n=4 total), stratified as 25% (n=1/4) in patients with major bleeding versus 3.7% (n=3/81) in those without major bleeding. Major bleeding significantly increased 30-day mortality risk (p=0.02). Time to death among the 9 deceased patients was <30 days (44.4%, n=4), 30-100 days (33.3%, n=3), and >100 days (22.2%, n=2). Kaplan-Meier analysis showed early survival divergence (Figure 4), with significantly lower survival in patients with major bleeding (n=4) compared to those without (n=81): 75% versus 96.3% at 30 days, and further divergence noted at 100 days (log-rank p<0.001).

## Discussion:

This retrospective cohort study, conducted at Uttar Pradesh University of Medical Sciences (UPUMS), Saifai, Etawah, India, from December 2024 to April 2025, examined transfusion requirements and bleeding risks in patients with acute coronary syndrome (ACS) receiving anticoagulation or thrombolytic therapy. Major bleeding occurred in 4.7% of patients, consistent with rates below 5% reported in prior studies of similar populations [6, 7]. This finding indicates that bleeding remains a persistent complication in ACS management despite therapeutic advances, highlighting the need for careful risk assessment and monitoring. Gastrointestinal bleeding was the most common site, comprising 50% of major events, in agreement with Manoukian [12], who identified the gastrointestinal tract as the predominant bleeding source in ACS patients treated with antithrombotic agents. This pattern likely reflects the combined effects of antiplatelet, anticoagulant and fibrinolytic therapies on mucosal integrity, particularly in patients with preexisting conditions such as peptic ulcer disease or gastritis. These results support routine screening for gastrointestinal risk factors (*e.g.*, history of ulcers or NSAID use) before initiating intensive antithrombotic therapy. Independent risk factors for major bleeding included age >65 years, chronic kidney disease (CKD) and concomitant use of antiplatelet, anticoagulant and fibrinolytic agents, aligning with established evidence [13]. Advanced age increases susceptibility through reduced drug clearance and greater vascular fragility [14], while CKD impairs platelet function and prolongs pharmacodynamic effects [20]. Triple therapy, administered to 30% of patients in this cohort, markedly elevated bleeding risk, illustrating the trade-off between ischemic protection and hemorrhagic safety [5]. Transfusion requirements were dominated by packed red blood cells (PRBCs), with a mean of 2 units per major bleeding event, consistent with patterns observed in ACS-related hemorrhage [8]. This underscores the value of structured blood conservation strategies to address anemia while minimising transfusion-associated complications such as transfusion-related acute lung injury or alloimmunisation [9]. Kaplan-Meier analysis revealed a significant adverse prognostic impact of major bleeding. Patients with major bleeding (n=4) exhibited lower 30-day survival (75.0% versus 96.3%) and 100-day survival (50.0% versus 90.1%) compared with those without bleeding (n=81; log-rank p<0.001). Early divergence in survival curves supports the view that major bleeding independently predicts mortality in ACS, likely through hemodynamic compromise or interruption of essential antithrombotic therapy [18, 21]. These findings reinforce the importance of individualised management, including dose reduction in elderly or CKD patients and preferential use of direct thrombin inhibitors (bivalirudin) in high-risk cases [15]. Study limitations include the retrospective, single-centre design and modest sample size (n=85), which may constrain generalisability and introduce selection bias or incomplete data capture [3]. Prospective, multicentre studies are warranted to improve external validity, validate bleeding risk stratification tools (CRUSADE score) [22] and evaluate gastroprotective strategies such as proton pump inhibitors [23].

## Conclusion:

We show that persistent challenge of bleeding complications in ACS management, with a 4.7% prevalence of major bleeding, predominantly gastrointestinal, driven by identifiable risk factors such as advanced age, CKD and combined antithrombotic therapy. The significant mortality impact of bleeding necessitates individualized treatment approaches and robust risk assessment tools to Optimize outcomes.

## Data availability:

The data that support the findings of this study are available on request from the corresponding author. The data are not publicly available due to privacy or ethical restrictions.

## Funding statement:

This research received no specific grant from any funding agency in the public, commercial, or not-for-profit sectors.

## Figures and Tables

**Table 1:** Patient demographics and medical history

**Characteristic**	**Number (n)**	**Percentage (%)**
Age >65 years	34	40
Male	52	61.2
BMI >25 kg/m^2^	45	52.9
Diabetes Mellitus	28	32.9
Hypertension	50	58.8
Chronic Kidney Disease	12	14.1
Liver Disease	5	5.9
Prior ACS	15	17.6
Smoking	30	35.3
Alcohol Use	20	23.5
Prior Anticoagulation/Antiplatelet	25	29.4

**Table 2:** Presenting symptoms

**Symptom**	**Number (n)**	**Percentage (%)**
Chest Pain	78	91.8
Shortness of Breath	45	52.9
Fatigue	30	35.3
Palpitations	15	17.6
Others (*e.g.*, nausea)	10	11.8

**Table 3:** ECG changes

**ECG Finding**	**Number (n)**	**Percentage (%)**
ST-Segment Elevation	40	47.1
ST-Segment Depression	25	29.4
T-Wave Inversion	15	17.6
Q Waves	3	3.5
None	2	2.4

**Table 4:** Percutaneous Coronary Intervention (PCI)

**PCI Performed**	**Number (n)**	**Percentage (%)**
Yes	60	70.6
No	25	29.4

**Table 5:** Bleeding event details

**Incidence**	**Number (n)**	**Percentage (%)**
**Bleeding Event**		
Yes	11	12.9
No	74	87.1
**Timing of Bleeding**		
< 24 hours	3	27.3
24 hours - 5 days	5	45.5
> 5 days	3	27.3
**Source of Bleeding**		
Gastrointestinal	5	45.5
Epistaxis	2	18.1
Access Site	3	27.3
Intracranial	1	9.1
**Source of Major Bleeding**		
Gastrointestinal (GI)	2	50
Intracranial	1	25
Access Site (Femoral)	1	25
**Severity of Bleeding**		
Minor (GI or Access Site, Supportive Care Only)	4	36.4
Moderate (GI, IV Fluid)	3	27.2
Major (Organ, *e.g.*, Intracranial, Transfusion)	4	36.4
**Transfusion Required**		
Yes (Major Bleeds Only)	4	36.4
No Minor and Moderate Bleeds)	7	63.6
**Type of Transfusion (Major Bleeds Only)**		
Packed Red Blood Cells (PRBC)	6	66.7
Platelets	2	22.2
Cryoprecipitate	0	0
Fresh Frozen Plasma (FFP)	1	11.1
Units Transfused (Major Bleeds Only)	Mean: 2 units (range: 1-4)	

**Table 6:** Multivariate logistic regression analysis of independent predictors of major bleeding in ACS patients

**Risk Factor**	**Odds Ratio (OR)**	**95% Confidence Interval (CI)**	**p-value**
Age > 65 years	4.8	1.6 - 14.2	<0.01
Chronic Kidney Disease (CKD)	6.1	2.0 - 18.7	<0.01
Triple Antithrombotic Therapy	5.5	1.8 - 16.9	<0.01
*p-value<0.05 significant

**Table 7:** Mortality outcomes with bleeding stratification

**Outcome**	**Number (n)**	**Percentage (%)**
Overall Survival	76	89.4
Overall Mortality	9	10.6
**30-Day Mortality**		
- With Major Bleeding (n=4)	1	25
- Without Major Bleeding (n=81)	3	3.7
- Total 30-day deaths	4	4.7
**Time to Death (n=9 deceased)**		
< 30 days	4	44.4
30-100 days	3	33.3
> 100 days	2	22.2
